# Can dedicated emergency team and area for older people reduce the hospital admission rate? - An observational pre- and post-intervention study

**DOI:** 10.1186/s12877-021-02044-w

**Published:** 2021-02-10

**Authors:** Jenny Liu, Therese Palmgren, Sari Ponzer, Italo Masiello, Nasim Farrokhnia

**Affiliations:** 1 Department of Clinical Science and Education, Södersjukhuset, Karolinska Institutet, Södersjukhuset AB, KI SÖS, Sjukhusbacken 10, 118 83 Stockholm, Sweden; 2 Department of Computer Science and Media Technology, Linnaeus University, Växjö, Sweden

**Keywords:** Emergency department, Older patients, Hospital admission, Length of stay, Interprofessional, Teamwork

## Abstract

**Background:**

Emergency department (ED) care of older patients is often complex. Geriatric ED guidelines can help to meet this challenge. However, training requirements, the use of time-consuming tools for comprehensive geriatric assessment (CGA), a lack of golden standard to identify the frail patients, and the weak evidence of positive outcomes of using CGA in EDs pose barriers to introduce the guidelines. Dedicating an interprofessional team of regular ED medical and nursing staff and an older-friendly ED area can be another approach. Previous studies of geriatrician-led CGA in EDs have reported a reduced hospital admission rate. The aim of this study was to investigate whether a dedicated interprofessional emergency team also can reduce the hospital admission rate without the resources required by the formal use of CGA.

**Methods:**

An observational pre-post study at a large adult ED, where all patients 80 years or older arriving on weekdays in the intervention period from 2016.09.26 to 2016.11.28 and the corresponding weekdays in the previous year from 2015.09.28 to 2015.11.30 were included.

In the intervention period, older patients either received care in the geriatric module by the dedicated team or in the regular team modules for patients of mixed ages. In 2015, all patients received care in regular team modules. The primary outcome measure was the total hospital admission rate and the ED length of stay was the secondary outcome measure.

**Results:**

We included 2377 arrivals in the intervention period, when 26.7% (*N* = 634) received care in the geriatric module, and 2207 arrivals in the 2015 period. The total hospital admission rate was 61.7% (*N* = 1466/2377) in the intervention period compared to 64.8% (*N* = 1431/2207) in 2015 (*p* = 0.03). The difference was larger for patients treated in the geriatric module, 51.1% compared to 62.1% (95% CI: 56.3 to 68.0%) for patients who would have been eligible in 2015. The ED length of stay was longer in the intervention period.

**Conclusions:**

An interprofessional team and area dedicated to older patients was associated to a lower hospital admission rate. Further studies are needed to confirm the results.

## Background

The care of older patients in the emergency department (ED) is often complex due to comorbidity, polypharmacy [1], cognitive and functional decline. In addition, older patients are frequent visitors who often present atypical symptoms, require more extensive diagnostic workup, stay longer in the ED, and are more often admitted to inpatient care [2–5]. Research on innovative care models for older ED patients is therefore a high priority [6]. Geriatric EDs incorporating various components of staff training, screening tools, and post-ED resources have evolved [7–9]. Collaborating medical and nursing associations have endorsed guidelines recommending older-friendly ED environments, screening of all geriatric patients for high risk of adverse outcomes, and components of a comprehensive geriatric assessment (CGA) [10, 11]. The American College of Emergency Physicians (ACEP) has launched an accreditation program, where EDs can chose to implement these ED geriatric guidelines at three different levels [12].

CGA is performed by interdisciplinary teams using multidimensional screening tools to assess medical, social and functional needs [13]. For older patients admitted to acute care wards designated for CGA the evidence of being alive and living at home is robust, but it is inconclusive in the ED setting [14–16]. Some studies of consultant geriatrician-led CGA in EDs have reported reduced hospital admission rates [17]. However, CGA is time consuming and should be reserved for frail ED patients, who need to be identified by a validated screening tool [18]. Although a consensus group has defined physical frailty [19], a lack of consensus on its operational definition [20–22], poor agreement between different frailty scores [23], and no gold standard for screening frail ED patients [24–28] persists. This may be a barrier to acquire the additional resources for ED staff training and geriatric interventions.

Healthcare teams improve the quality of care and patient safety [29–32] and the principles of efficient teamwork in EDs have been summarized by researchers [33, 34]. We have previously reported that interprofessional teamwork in an ED reduced the ED length of stay (LOS) compared to two common triage strategies [35]. We lacked resources to implement the geriatric ED guidelines or CGA but were able to dedicate one interprofessional team and an older-friendly area to improve the care for older patients. The aim of the study was to evaluate this pilot project and our research question was: Can a dedicated emergency team and area for older people reduce the hospital admission rate?

## Methods

This was an observational before-and-after study conducted at Södersjukhuset, a 600-bed urban teaching hospital with 110,000 adult ED visits per year in Stockholm, Sweden. Patients 80 years or older accounted for 15% of the visits, although they only constitute 5% of the Swedish population. Most patients presented to the Swedish EDs without first seeing a general practitioner. Like many acute hospitals, this hospital had only acute care wards and no geriatric wards. This meant that older patients were transferred for geriatric inpatient care after an acceptance for admission by the receiving hospitals.

### Study population & periods

We included all ED visits by patients 80 years or older arriving during 45 consecutive weekdays of the project from 2016.09.26 to 2016.11.28 and the corresponding weekdays in the previous year from 2015.09.28 to 2015.11.30 with only regular emergency teams for adult patients of mixed ages. The project was not staffed on Friday 4 Nov 2016, we therefore excluded this day and the corresponding Friday 6 Nov 2015. The lack of consensus on a standard geriatric age limit has caused previous studies to use a wide range of cut-off ages from 60 to 85 years [17, 18, 36]. Considering the high level of independence among Swedish people 60 years or older, we included the oldest age category, 80 years or older, of the yearly report of the Swedish National Board of Health and Welfare (Socialstyrelsen). The ED LOS of this age group is also a national quality measure [5].

### Intervention

During the intervention period in 2016, an interprofessional team of regular ED medical and nursing staff was dedicated to the geriatric module, a calmer area where ten hospital beds replaced ED gurneys and hot food was available. The staff received no special geriatric training and CGA tools were not introduced. This geriatric module operated from Monday 8 am to Friday 3:30 pm. It was staffed by an emergency physician or a senior resident, an intern, a registered nurse, two nursing assistants, and a specialist nurse from 8 am to 9 pm, but only a registered nurse and a nursing assistant during night shifts. The specialist nurse had expertise in discharge planning for older patients and networked with local geriatric hospitals. Such specialist nurses were also available for the regular ED during both study periods, but as consultants rather than team members. They had a case management approach by focusing on the older patients’ need for more care or service at home and facilitated the transition to geriatric or primary care. However, they did not have a standardized approach to evaluate the older patients, for example, with regard to fall risk or cognitive function. The interprofessional work process was otherwise similar in the geriatric module and the regular ED during both periods and has been described in our previous studies [35, 37].

All patients 80 years or older were eligible for the geriatric module, but younger patients 65 to 79 years old with comorbidities could also be accepted by the geriatric team. High-acuity patients arriving with prehospital alert or needing continuous monitoring of unstable vital signs were excluded, because the older-friendly area lacked the necessary equipment. The excluded patients would have been assigned the red or orange acuity level if the 5-level Rapid Emergency Treatment and Triage System (RETTS) [38] had been in use. However, the triage teams and RETTS had been replaced, when interprofessional teamwork was introduced in November 2014. Instead of assigning each patient an acuity level, a senior nurse in each teamwork module was responsible for the queueing patients and communicated high-priority patients to the other team members [35]. This senior nurse recruited older patients from the registration or the other teamwork modules, when space became available in the geriatric module. This module was estimated to enrol two new geriatric patients per hour between 8 am and 9 pm. The capacity was not enough to enrol all eligible patients, especially during peak hours.

### Outcome measures and data collection

The primary outcome measure was the combined proportion of patients admitted to acute care wards at the study hospital and those transferred to receiving hospitals. The secondary outcome measure was the ED LOS, measured as the time interval from registration at ED arrival to departure. We retrospectively collected de-identified patient data from the electronic ED registry and retrieved the variables age, sex, arrival mode, chief complaint and disposition of the patients. The time of arrival and departure were also extracted to calculate the ED LOS. From the hospital bed occupancy registry, we collected data of available in-hospital beds and the number of inpatients at 6 am each weekday during the study periods.

### Statistical analysis

The data was imported to R version 3.2.4 (The R Foundation for Statistical Computing, Vienna) and IBM SPSS Statistics version 26 for statistical analysis. We used Pearson’s χ^2^ test to compare proportions and the Mann-Whitney-Wilcoxon test to compare mean values. Since the distribution of ED LOS is heavily skewed with a short LOS for most patients and few with very long LOS, we used median values to compare the study groups and obtained 95% confidence intervals (CI) by bootstrap sampling. We also used bootstrap sampling to simulate a 2015 group with the same chief complaints and arrival modes as that of the geriatric module in 2016.

We used linear regression analyses to explore the differences in patient and background characteristics between the study periods, with the ED LOS as the dependent variable. We checked that that each model met the normality and homogeneity assumptions and ruled out collinearity. The statistical significance level was set at the two-tailed *p*-value of 0.05 for all outcome measures.

## Results

**Fig. 1:**
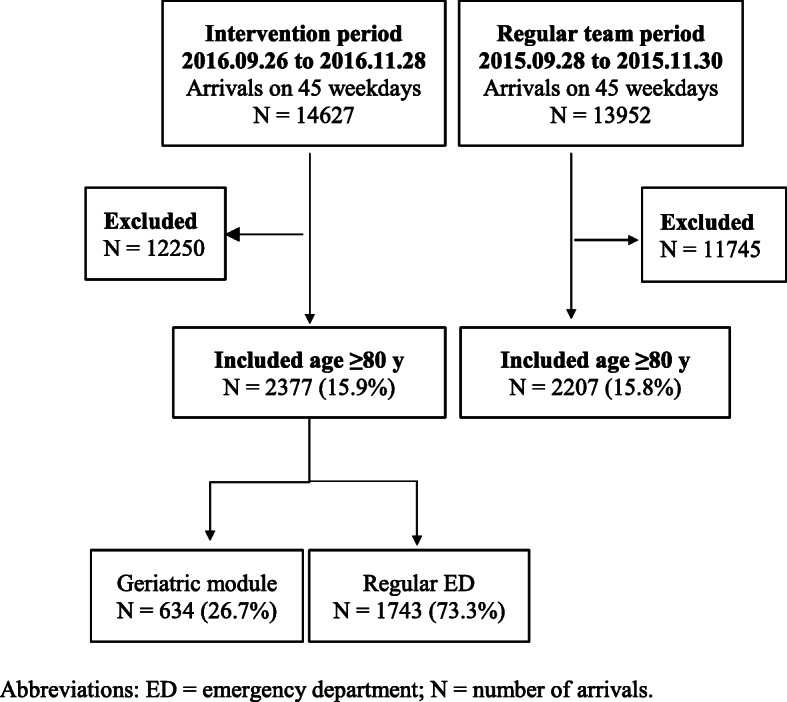
Flow diagram of the study population. Patients 80 years or older arriving to the emergency department (ED) on weekdays in the intervention period 2016 and the corresponding days in the 2015 period were included. In the intervention period, older patients either received care in the geriatric module, or in the regular ED

In the period with only regular team modules from 2015.09.28 to 2015.11.30, we included 2207 arrivals by patients 80 years or older from a total of 13,952 adult arrivals. From 14,627 adult arrivals in the intervention period from 2016.09.26 to 2016.11.28, we included 2377 arrivals by patients 80 years or older. Of these, 634 (26.7%) patients received care in the geriatric module and the remaining 1743 (73.3%) in the regular ED (Fig. 1).

During the intervention period in 2016, 948 patients were treated in the geriatric module with a mean arrival rate ranging from 1.7 to 2.3 patients per hour from 8 am to 9 pm. Of these, 634 patients were 80 years or older with a mean hourly arrival rate ranging from 1.3 to 1.9. The hourly arrival rate of all 2377 patients 80 years or older reached a maximum of 5.3 at 1 pm, which exceeded the estimated capacity of two patients per hour in the geriatric module.

### Patient characteristics

The proportion of patients 80 years or older and the distribution of age, sex, arrival mode, and chief complaint were similar in both periods. In the intervention period, the distribution of arrival mode and chief complaint differed between the geriatric module and regular ED. High-acuity patients received care in the regular ED, where the most common chief complaints were breathing problem, neurological deficit and chest pain. By contrast, patients presenting head injury, abdominal pain and malaise were more likely to receive care in the geriatric module, where a larger proportion arrived by ambulance without prehospital alert (Table 1).

**Table 1:** Patient characteristics

	2016 intervention period			2015 period
	Geriatric module	Regular ED	Total	Total
**Arrivals age ≥ 80 years,** N	634	1743	2377	2207
**Mean age,** years (SD)	86.7 (4.7)	86.4 (4.7)	86.4 (4.7)	86.6 (4.6)
**Sex**	**N (%)**	**N (%)**	**N (%)**	**N (%)**
Male	258 (40.7)	683 (39.2)	941 (39.6)	871 (39.5)
Female	376 (59.3)	1060 (60.8)	1436 (60.4)	1336 (60.5)
**Arrival mode**				
Prehospital alert	1 (0.2)	235 (13.5)	236 (9.9)	239 (10.8)
Ambulance/helicopter	370 (58.4)	902 (51.7)	1272 (53.5)	1186 (53.7)
Other	263 (41.5)	606 (34.8)	869 (36.6)	782 (35.4)
**Top 10 chief complaints**				
Breathing problem/dyspnoea	41 (6.5)	232 (13.3)	273 (11.5)	287 (13.0)
Chest pain	22 (3.5)	163 (9.4)	185 (7.8)	190 (8.6)
Hip injury	37 (5.8)	121 (6.9)	158 (6.6)	163 (7.4)
Head injury	59 (9.3)	95 (5.5)	154 (6.5)	140 (6.3)
Limb swelling/pain	46 (7.3)	94 (5.4)	140 (5.9)	159 (7.2)
Abdominal pain	50 (7.9)	81 (4.6)	131 (5.5)	159 (7.2)
Stroke/neurological deficit	13 (2.1)	97 (5.6)	110 (4.6)	119 (5.4)
Malaise	41 (6.5)	50 (2.9)	91 (3.8)	133 (6.0)
Arrhythmia	2 (0.3)	91 (5.2)	93 (3.9)	65 (2.9)
Vertigo	28 (4.4)	36 (2.1)	64 (2.7)	74 (3.4)

Patients 80 years or older arriving to the emergency department (ED) on 45 weekdays in the intervention period from 2016.09.26 to 2016.11.28 and the corresponding weekdays from 2015.09.28 to 2015.11.30 were included. In the intervention period, these patients either received care in the geriatric module, or in the regular ED. Abbreviations: *ED* Emergency department, *N* Number, *SD* standard deviation

### Background characteristics

The mean daily ED volume was larger in the intervention period, 335.0 (SD 27.1) arrivals compared to 321.5 (SD 27.3) in 2015 (*p* < 0.01, 95% CI: 11.9 to 15.0). The hospital’s mean in-bed occupancy rate for the wards receiving patients from the adult ED was higher in the intervention period, 99.1% (SD 2.8) compared to 92.8% (SD 3.0) in 2015 (*p* < 0.01, 95% CI: 5.1 to 7.6%). This was a combined effect of a larger number of in-patients in 2016, mean 452 (SD 14.1) compared to 434 (SD 13.7) in 2015, and fewer in-beds in 2016, mean 461 (SD 7.1) compared to 471 (SD 6.9) in 2015. The number of in-beds was reduced from June 2016, when the emergency department closed its 10-bed Observation ward. In the 2015 period, 55 patients 80 years or older were admitted to this Observation ward. Of these, 13 patients were moved to an acute care ward, 11 were transferred to receiving hospitals, and 31 were discharged home. All patients left the ward on the same day or the day after admission, except one who was moved to an acute care ward after 2 days.

### Outcome measures

A smaller proportion of the included patients was admitted to acute care wards at the study hospital in the intervention period, 45.2% (*N* = 1074) compared to 50.8% (*N* = 1121) in 2015 (*p* < 0.01). At the same time, a larger proportion of the patients was transferred to receiving hospitals in the intervention period, 16.5% (*N* = 392) compared to 14.0% (*N* = 310) in 2015 (*p* = 0.02). This means that the combined proportion of patients admitted to acute care wards and those transferred to receiving hospitals was lower in the intervention period, 61.7% compared to 64.8% in 2015 (*p* = 0.03). In addition, a larger proportion of the patients was discharged to home in the intervention period, 36.7% (*N* = 872) compared to 33.8% (*N* = 745) in the 2015 period (*p* = 0.04) (Table 2).

**Table 2:** Outcome measures

	2016 intervention period			2015 period	***p***
	Geriatric module	Regular ED	Total	Total	
**Arrivals,** N	634	1743	2377	2207	
**ED LOS,** min					
Median (95% CI)	390 (378–407)	313 (304–320)	330 (322–337)	275 (267–283)	< 0.01
**ED disposition**	**N (%)**	**N (%)**	**N (%)**	**N (%)**	
Admitted to acute care wards	198 (31.2)	876 (50.3)	1074 (45.2)	1121 (50.8)	< 0.01
Transferred to receiving hospitals	126 (19.9)	266 (15.3)	392 (16.5)	310 (14.0)	0.02
Discharged to home	306 (48.3)	566 (32.5)	872 (36.7)	745 (33.8)	0.04
Other	4 (0.6)	35 (2.0)	39 (1.6)	31 (1.4)	0.51

Emergency department (ED) length of stay and dispositions for patients 80 years or older in the intervention period from 2016.09.26 to 2016.11.28 and the corresponding days from 2015.09.28 to 2015.11.30. In the intervention period, these patients either received care in the geriatric module, or in the regular ED. Abbreviations: *CI* Confidence interval, *ED* Emergency department, *N* Number; *LOS* Length of stay, *SÖS* Södersjukhuset

The median ED LOS was longer in the intervention period, 330 min (95% CI: 322 to 337) compared to 275 min (95% CI: 267 to 283) in the 2015 period. However, differences in background characteristics could have influenced the ED LOS. We used multi-variate linear regression analysis to explore these differences, in which we also included patient age, sex and arrival mode as predictor variables. The analysis indicated 84 min longer ED LOS for patients treated in the geriatric module (Table 3).

**Table 3:** ED length of stay (minutes) as the dependent variable in linear regression analysis

Predictor variable	Range	Beta	Std Error	Sig.
(Constant)		−62.833	126.981	0.621
Age (years)	80 to 107	−0.090	0.654	0.891
Sex	Female = 1, Male = 0	12.469	6.145	0.043
Arrival by ambulance/helicopter				
With prehospital alert	Yes = 1, No = 0	−31.875	10.655	0.003
Without prehospital alert	Yes = 1, No = 0	83.383	6.538	< 0.001
Daily ED volume (Number of arrivals)	261 to 400	0.717	0.113	< 0.001
Daily hospital in-bed occupancy	0.662 to 1.050	102.182	109.141	0.349
Geriatric module	Yes = 1, No = 0	84.024	9.483	< 0.001
Period	2016 = 1, 2015 = 0	34.978	9.781	< 0.001

Abbreviation: *ED* Emergency department

To compare patients in the geriatric module in 2016 to those who would have been eligible if the geriatric module had been introduced in 2015, we simulated 10,000 bootstrap samples. Each sample consisted of 634 patients from the 2015 period with the same distribution of chief complaint and arrival mode as those of the geriatric module in 2016. In the bootstrap samples, 44.1% (95% CI: 39.5 to 48.8%) of the patients were admitted to acute care wards and 18.0% (95% CI: 14.5 to 21.7%) were transferred to receiving hospitals, which means a combined admission rate of 62.1% (95% CI: 56.3 to 68.0%). The proportion of patients discharged to home was 37.2% (95% CI: 32.8 to 41.6%) and the median ED LOS for all patients in the bootstrap samples was 291 min (95% CI: 227 to 307).

## Discussion

This study evaluated a geriatric intervention, where an interprofessional team of ED medical and nursing staff and an older-friendly area were dedicated to older patients. Our main finding was a lower total hospital admission rate for all patients 80 years or older during the intervention period, 61.7% compared to 64.8% during the corresponding period in the previous year. The difference was larger for patients treated in the geriatric module, 51.1% compared to 62.1% for patients with the same distribution of chief complaint and arrival mode in 2015.

The arguments for reducing the hospital admissions of older patients are not merely economical. For them, acute hospitalization is a major risk by causing an irreversible decline of the functional status, in addition to the risks of complications and adverse events [39, 40]. Studies of inappropriate hospital admissions have reported that a significant proportion of the older patients could have received care at lower levels than the acute hospitals [41, 42], and that the social circumstances surrounding the patient influence the physician’s decision to admit [43]. This means that some hospital admissions are avoidable by addressing social barriers to be discharged home or by arranging follow-up care. This was even suggested as the primary value of CGA in the ED by the authors of a systematic review of consultant geriatrician-led CGA in the ED [17]. They argued that CGA facilitates the ED teams to safely discharge complex patients who would otherwise have required hospital admission. In these studies, the reduction of hospital admissions ranged from 2.4 to 8.4 percentage points [44–46] and the same day discharge rate increased from 1.4 to 17.1% [47].

More recent studies of EDs operationalizing the geriatric ED guidelines have been published, where CGA conducted by transitional care nurses in three EDs reduced hospital admission rates by 5, 10, and 17 percentage points, respectively [48]. Another similar Geriatric ED intervention increased the likelihood of discharge with a hazard ratio of 1.2 [49]. These nurses operated as CGA specialists assisting the primary ED staff, and one may assume that the varying effect in these studies depended on the extent CGA results influenced the decision-making of the primary ED staff. In contrast, the specialist nurse was a team member of the geriatric module, enabling a close collaboration and sharing of goals for individual patients. Older patients competed with younger and high-acuity patients in the regular ED, whereas in the geriatric module the staff could focus on the complex needs of older patients.

The median ED LOS is seldom reported in geriatric studies. Since CGA is time consuming, one may expect a longer ED LOS. To our knowledge, only one paper has reported a shorter ED LOS with hazard ratios 1.28 to 1.48 [49].

## Limitations

This study has several limitations. Patients were not randomised, which may have introduced bias when selecting patients to the geriatric module. To account for this, we compared all patients 80 years or older in the intervention period to those in the previous year. A before-and-after design may not claim a causality between the intervention and the outcomes. The results from this single centre may not be transferable or generalisable to different ED settings.

## Conclusions

This study investigated an interprofessional emergency team and area dedicated to older people, a novel approach which may be considered as a first step to implement the geriatric ED guidelines. We found an association between the geriatric module intervention and a lower hospital admission rate, thereby avoiding the risk for functional decline, complications and adverse events associated to hospitalization. However, future randomised controlled studies are needed, preferably involving multiple centres and including patient experience and functional status as outcome measures.

## Data Availability

The datasets used during the current study are available from the following public repository: https://figshare.com/articles/dataset/Geriatric_module_in_an_ED/13107281. 10.6084/m9.figshare.13107281.

## Abbreviations

CGA: Comprehensive geriatric assessment
CI: Confidence interval
ED: Emergency department
LOS: Length of stay
min: Minutes
N: Number
SD: Standard deviation
SE: Standard error
